# Metal–Organic Frameworks for Cisplatin Delivery to Cancer Cells: A Molecular Dynamics Simulation

**DOI:** 10.1021/acsomega.4c01437

**Published:** 2024-04-22

**Authors:** Elham Mashayekh, Zahra Nouri Khajeh Ghiasi, Iman Bhia, Zohreh Arefi Khorrami, Omid Malekahmadi, Mohammed Bhia, Samira Malekmohammadi, Yavuz Nuri Ertas

**Affiliations:** † Department of Immunology, Faculty of Medical Sciences, Tarbiat Modares University, Tehran 14115, Iran; ‡ Department of Chemical Engineering, Islamic Azad University, Shahrood Branch, Shahrood 36155163, Iran; § Faculty of Medicine, Shahid Beheshti University of Medical Sciences, Tehran 1985717443, Iran; ∥ Department of Chemical Engineering, Amirkabir University of Technology (Tehran Polytechnic), 424 Hafez Avenue, Tehran 1591634311, Iran; ⊥ Department of Mining and Metallurgical Engineering, Yazd University, Yazd 89195, Iran; # Department of Pharmaceutics and Pharmaceutical Nanotechnology, School of Pharmacy, Shahid Beheshti University of Medical Sciences, Tehran 1996835113, Iran; ∇ School of Materials, University of Manchester, Engineering Building A, MECD, Manchester M1 3BB, U.K.; ○ ERNAM−Nanotechnology Research and Application Center, Erciyes University, Kayseri 38039, Türkiye; ◆ Department of Biomedical Engineering, Erciyes University, Kayseri 38039, Türkiye

## Abstract

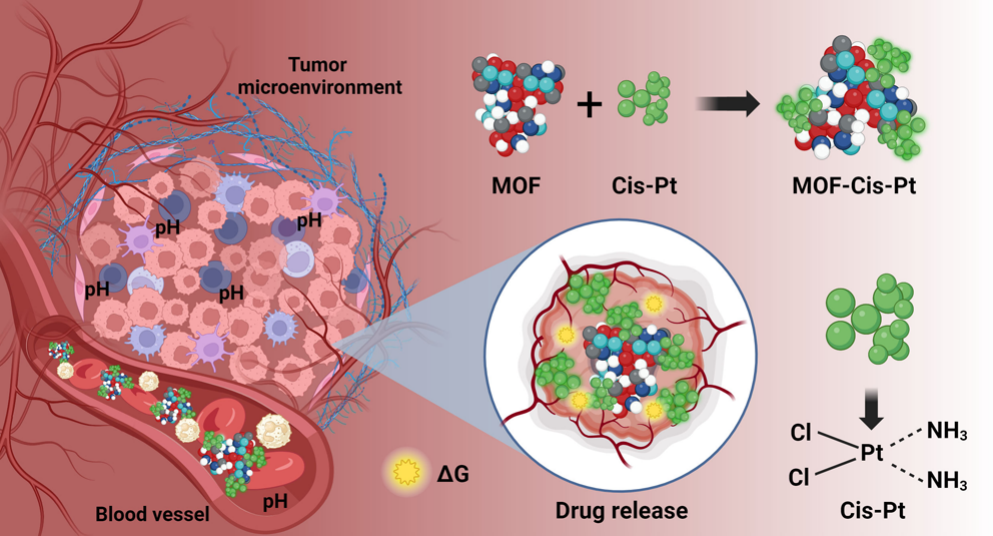

Metal–organic frameworks (MOFs) are utilized as nanocarriers to enhance the efficiency of chemotherapy drugs, including cisplatin, which exhibit limitations such as side effects and resistance mechanisms. To evaluate the role of MOFs, we employed a molecular dynamics simulation, which, unlike other experiments, is cost-effective, less dangerous, and provides accurate results. Furthermore, we conducted molecular docking simulations to understand the interaction between cisplatin and MOF, as well as their internal interactions and how they bind to each other. Cisplatin and MOF molecules were parametrized using the Avogadro software and x2top command in GROMACS 5.1.2 and optimized by CP2K software; the Charmm-GUI site parametrized the cell cancer membrane. Three molecular dynamics simulations were conducted in four stages at various pHs, followed by simulated umbrella sampling. The simulations analyzed the pH responsiveness, total energy, Gibbs free energy, gyration radius, radial distribution function (RDF), solvent accessible surface area, and nanoparticles’ toxicity. Results demonstrated that a neutral pH level (7.4) has greater adsorption and interaction compared to acidic pH values (6.4 and 5.4) because it displays the highest total energy (−17.1 kJ/mol), the highest RDF value (6.66), and the shortest distance (0.51 nm). Furthermore, the combination of cisplatin and MOFs displayed increased penetration compared to that of their individual forms. This study highlights the suitability of MOFs as nanocarriers and identifies the optimal pH values for desirable outcomes. Thus, it provides future studies with appropriate data to conduct their experiments in assessing MOFs.

## 1 Introduction

Cisplatin, (SP-4–2)-diamminedichloridoplatinum(II), or cisplatinum (CAS No. 15663–27–1, MF-Cl2H6N2Pt; NCF-119875), is a cytotoxic agent used in various cancers as monotherapy or as part of a combination chemotherapy or radiation therapy.^1,2^ Cisplatin was first synthesized by Peyrone in 1845.^3^ Its properties were fortuitously discovered in 1965 by Dr. Barnett Rosenberg while observing electric current effects on bacterial growth.^4,5^ Rosenberg reported that electric fields lead to filamentous growth of *Escherichia coli* cells.^4,5^ Since filamentation indicates deoxyribonucleic acid (DNA) cell damage, further studies were conducted to assess the biological activities of platinum complexes.^4^ It was discovered that cisplatin inhibits the growth of tumor cells such as leukemia L1210 cells and sarcoma 180 cells in mice,^6,7^ the Walker 256 carcinosarcoma,^8^ methylbenzanthracene-induced mammary carcinoma,^9^ and Dunning ascitic leukemia in rats.^10^ Finally, after conducting clinical trials, cisplatin was FDA-approved in 1978 under the name Platinol as an anticancer drug for testicular, ovarian, lung, and bladder cancers.^5^

Cisplatin is activated upon entering the cell’s cytoplasm as water molecules replace the chloride atoms of cisplatin, resulting in the formation of a highly reactive electrophile that can react with any nucleophile.^11,12^ The binding between cisplatin and a purine residue, specifically at the N7 reactive center, leads to apoptotic cell death, DNA damage, and inhibition of cell division.^2^ One of the most notable mechanisms underlying cisplatin toxicity is oxidative stress in the mitochondrion, which hampers calcium uptake, reduces mitochondrial membrane potential, and leads to the loss of sulfhydryl groups in mitochondrial proteins.^2^ Another important mechanism is calcium signaling, which triggers enzyme inhibition and lipid peroxidation, activating the extracellular signal-regulated kinase, resulting in cell cycle arrest in adenocarcinoma ovarian cell lines.^13^ Thus, it leads to Jun amino-terminal kinase pathway activation, causing DNA damage,^14^ and modulating cell apoptosis through p38 mitogen-activated protein kinase.^15,16^

Despite its anticancer effects, the use of cisplatin is restricted due to significant limitations, including its side effects, the development of resistance mechanisms, and poor bioavailability.^17,18^ The most frequently observed side effects are ototoxicity, hepatotoxicity, nephrotoxicity, myelosuppression, and cardiotoxicity due to the nonselective distribution of the drug between healthy and tumor tissue.^17,18^ Nonselective targeting and a low ability to differentiate between cancerous and normal cells are the main causes of cisplatin toxicity.^19^ Resistance is attributed to the decrease in drug accumulation in the cell, led by increased efflux and reduced influx.^20,21^ Also, it was reported that overexpression of ATP7A caused resistance in the esophagus,^22^ lung,^23^ cervical, and ovarian cancers.^21,23^ As for its physicochemical properties, cisplatin possesses a density of 3.74 g/cm^3^, molecular weight of 301.1 g/mol, a melting point of 270 °C, a water solubility of 2.53 g/L at 25 °C, and a log* K*_ow_ of −2.19,^2^ which indicates low lipophilicity and water solubility.^24,25^

In recent years, drug delivery systems (DDS) have played a crucial role in tackling challenges associated with anticancer treatments. These challenges include low bioavailability, solubility, permeability, toxicity, and retention effects.^26−28^ Various types of DDSs have been employed for delivering anticancer drugs effectively. For instance, nanosheets have been utilized to enhance the transportation of doxorubicin, resulting in a higher therapeutic dose.^29^ Nanotubes have provided increased stability for 5-fluorouracil,^30^ while liposomes have facilitated higher drug release in lomustine.^31^ Furthermore, metal–organic frameworks (MOFs) have been employed in delivering luteolin, platinum, and cisplatin for drug-resistant cancers.^32−35^

MOFs, also known as porous coordination polymers, possess diverse properties, such as being nanocarriers for chemotherapeutics and imaging contrast agents.^34,36−38^ MOF structures are characterized by their large pores and a surface composed of metal ions, allowing for the binding of desired components.^34^ Its distinctive characteristics include a high surface area, adjustable pore sizes, and variable chemical composition.^38^ Thus, MOFs can be utilized to overcome cisplatin limitations due to their selective targeting abilities and low toxicity, which may result in a higher therapeutic index.^38,39^ Initially, MOFs were employed for the codelivery of cisplatin and siRNA in ovarian cancer cells that exhibited resistance to the drug, which enhanced in vitro chemotherapeutic efficacy.^34^ Also, MOFs exhibit a distinctive characteristic of undergoing reversible structural changes in response to varying pH conditions. The organic ligands within pH-responsive MOFs incorporate functional groups capable of protonation or deprotonation in response to pH changes.^40,41^ This dynamic nature allows pH-responsive MOFs to selectively adsorb or release guest molecules, making them highly attractive for controlled drug delivery systems. In the realm of drug delivery, pH-responsive MOFs offer a promising strategy for improving drug efficacy while minimizing side effects.^42^ By incorporating pH-sensitive ligands, these MOFs remain stable in the neutral pH environment of the bloodstream and selectively release encapsulated drugs at target sites characterized by slightly acidic pH, such as tumor tissues.^41^ This pH-triggered drug release strategy enhances drug bioavailability and reduces off-target effects, offering a potential solution to combat drug resistance and enhance patient outcomes.^41^

However, MOFs are hampered by high production costs and low stability, posing challenges for their study as drug delivery systems.^39^ Moreover, there is a lack of data and research regarding MOFs’ permeation through cancer cell membranes at the molecular level. In order to overcome these difficulties, molecular dynamic simulations are considered a valuable method that provides quantitative and qualitative data regarding the physical-chemical mechanisms and interactions of MOFs as drug delivery systems.^43^ Moreover, simulations serve as a tool for predicting and assessing the performance and properties of different components, as well as the advantages of avoiding potential health risks associated with exposure to hazardous agents, such as anticancer drugs.^44^ The available evidence for the use of MOFs as nanocarriers is still insufficient and does not offer a clear picture of the delivery mechanisms to cancer cells. Thus, the current study aims to tackle this challenging gap by providing a molecular depiction of the uptake of MOFs loaded with cisplatin in cancer cells. Therefore, we will evaluate the effectiveness of MOFs as carriers for delivering cisplatin to cancer cells in a molecular dynamics simulation in a pH-responsive drug delivery system. Our simulation has thoroughly addressed all pertinent aspects concerning in silico investigations. Our analyses include the absorption and release of drugs at various pH levels, encompassing absorption during synthesis outside the body and release during drug interaction with cancer cells. Additionally, we have conducted other in silico investigations that provide comprehensive analyses of the passage of nanoparticles and drugs through cancer cell membranes.

## 2 Materials and Methods

The cancer cell membrane was constructed and parametrized using the Charmm-GUI site (http://www.charmm-gui.org). The membrane components consisted of sphingomyelin (SM), 1,2-dioleoyl-*sn*-glycero-3-phosphocholine (DOPC), 1,2-dioleoyl-*sn*-glycero-3-phospho-l-serine (DOPS), and 1,2-dioleoyl-*sn*-glycero-3-phosphoethanolamine (DOPE).^31−35^ We have opted for a simplified membrane model to streamline the cellular model and focus on the core issues.

**Figure 1:**
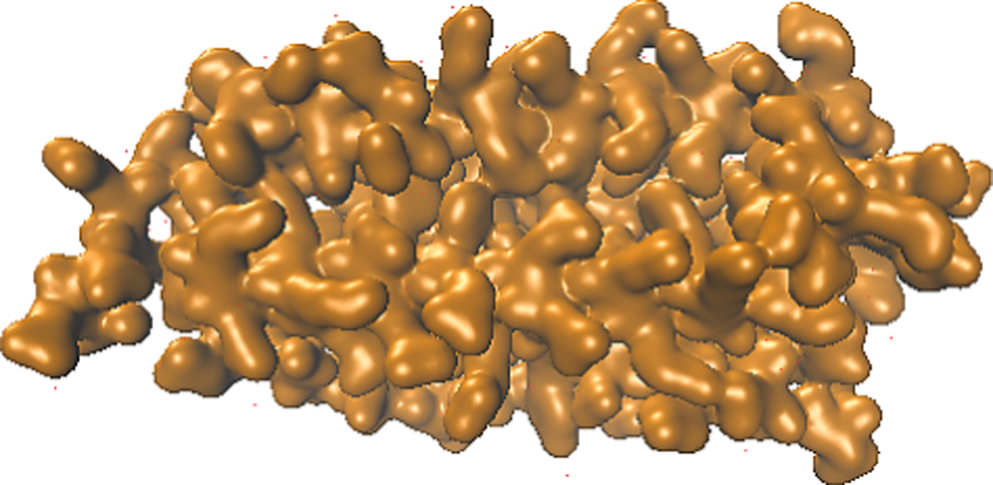
Representation of the porous structure of the MOF in the VMD software.

All simulations in this study were performed using GROMACS version 5.1.2 software, and VMD software was utilized as the graphical interface. OPLS-AA force field was employed, and the leapfrog algorithm was utilized for integrating Newton’s equations of motion. It is worth mentioning that thanks to the LINCS algorithm, all molecular dynamics simulations were conducted with a time step of 2 fs. In all molecular dynamics simulations, four main stages were carried out: energy minimization (EM), temperature equilibration (NVT), pressure equilibration (NPT), and the main run, molecular dynamics (MD). The energy minimization stage was performed using the steepest descent algorithm until the maximum force between two steps was less than 1000 kJ/mol/nm. In the two temperature equilibration stages, the V-rescale thermostat and Parrinello–Rahman barostat with time constants of 1 and 2 ps were utilized to stabilize the temperature and pressure at 300 K and 1 bar, respectively. In all of these simulations, a cutoff radius of 1 nm was considered for electrostatic and van der Waals interactions, employing the Verlet scheme for a duration of 10 ps. It is important to note that periodic boundary conditions were applied in all directions in the molecular dynamics simulations. To establish the initial configuration for molecular dynamics simulations, the initial structures of the Cisplatin and MOF molecules were drawn using the Avogadro software and we utilized the VMD software and represented the porosity in the MOF structure using the Volmap tool (Figure 1). The PolyParGen server and CP2K software were utilized for parametrizing the resulting structure. After applying the settings related to selecting the level of theory and their corresponding basis sets, which were B3LYP for the DFT function and 6-311++G, and applying the electrostatic potential (ESP), the topology file of the corresponding structure was generated by editing the output file from the PolyParGen server. Subsequently, the molecules were parametrized using the x2top command in GROMACS 5.1.2. Additional molecular information was obtained from the OBGMX server (http://software-lisc.fbk.eu/obgmx/). Finally, after constructing the simulation box measuring 6 × 6 × 6 cubic nanometers and solvating it, to investigate the drug release process, three simulations were conducted at different pH values (7.4, 6.4, and 5.4), and the four main stages of molecular dynamics simulation were carried out with a duration of 200 ns. In molecular dynamics simulations, we cannot alter the pH during the simulation. According to the Bronsted–Lowry theory, which is one of the fundamental concepts in chemistry regarding the behavior of acids and bases, any reaction involving the transfer of a proton from one substance to another is termed an acid–base reaction from the perspective of Bronsted–Lowry. According to this theory, an acid is a substance that donates a proton to another substance and a base is a substance that accepts a proton from another substance, and this holds true for hydrogen atoms. Now, pH, as named by Danish chemist S. P. L. Sørensen, is a scale that indicates the acidity or acidic properties of a solution to us. In molecular dynamics, we cannot use free hydrogen for pH changes. Instead, by adding or removing hydrogen atoms from each molecule, we apply pH changes to each molecule. Various software programs can be used for this purpose in molecular dynamics. We selected the Avogadro software. In this software, for the desired molecule, we change the pH range, and the molecule receives more hydrogen atoms in an acidic state (acquires a positive charge) and loses its hydrogen atoms in a basic state (acquires a negative charge). This method was used to apply pH to simulated molecules, and after adjusting the pH for each molecule, we parametrized them again.

### 2.1 Umbrella Sampling

After performing the primary simulation, “Umbrella Sampling” was carried out to compute the Gibbs free energy for membrane simulation. Umbrella Sampling is a molecular simulation method used to calculate the probability distribution or Gibbs free energy in a defined space. In this method, molecules are guided to specific states in the defined space or simulation box by using one or multiple energy belts (umbrella potential). Then, by performing molecular simulations in each state, the corresponding probability distribution is obtained. By combining these distributions using analytical methods such as WHAM (weighted histogram analysis), we can calculate the probability distribution or free energy for the entire defined space. Therefore, in this study, after conducting the primary simulation with a simulation box of dimensions 6 × 6 × 6, umbrella sampling was performed, which necessitates expanding the dimensions of the simulation box (to pull molecules into specific states in the defined space). Thus, the dimensions of the box were considered as 30 × 8 × 8 cubic nanometers.

In the three simulations, the MOF, MOF-Cis-Pt, and Cis-Pt molecules were exposed only superficially to the cancer cell membrane. The simulation was conducted over a duration of 300 ns with a time step of 2 fs. Initially, the simulation proceeded through four stages: energy minimization (EM), canonical ensemble (NVT), isothermal–isobaric ensemble (NPT), and molecular dynamics (MD). The output of the simulation was then used for umbrella sampling. During the umbrella sampling simulation, the cancer cell membrane was restrained, and the MOF, MOF-Cis-Pt, and Cis-Pt molecules were allowed to pass through the membrane using a pull code. These molecules were stretched by 12 nm along the *Z*-axis. Following the execution of the pull code, a total of 120 configurations were extracted at intervals of 0.1 nm. Finally, the Gibbs free energy was calculated by applying the weighted histogram analysis method (WHAM) to the configuration results.

### 2.2 Molecular Docking

**Figure 2:**
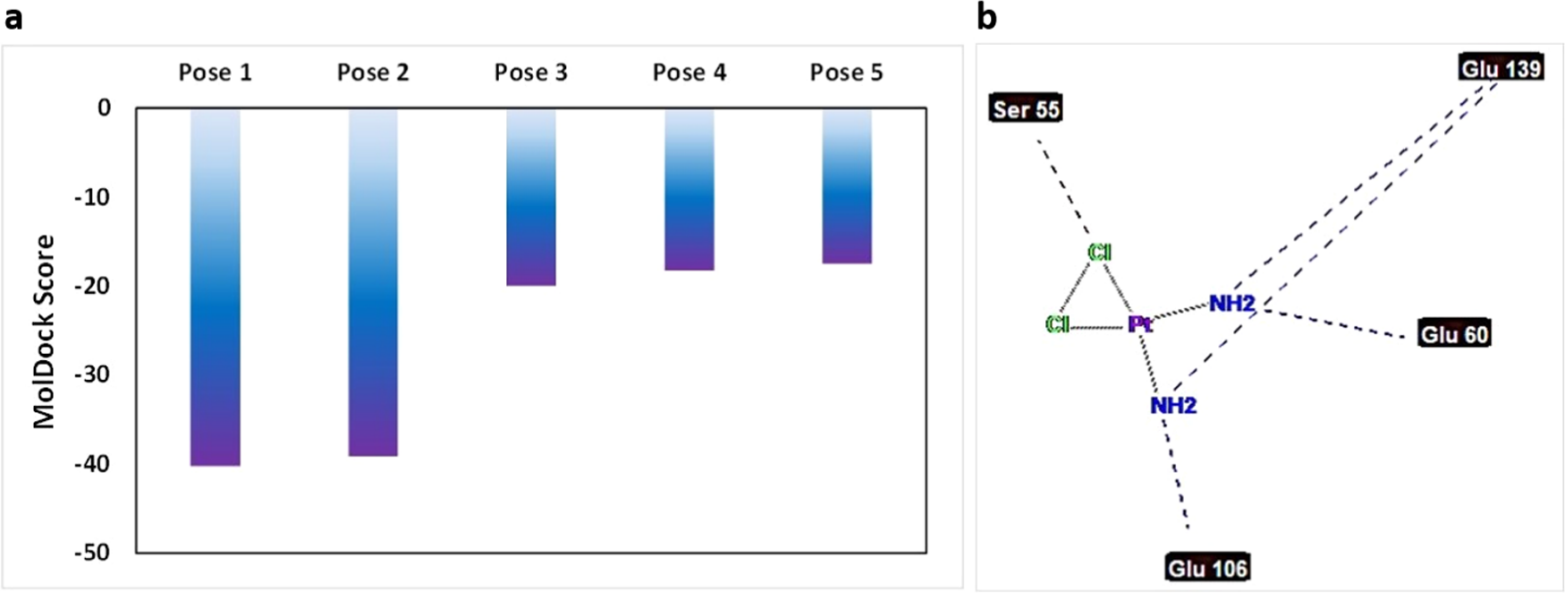
(a) MolDock Score at five poses, which is more negative in pose 1. (b) The interaction between cisplatin and MOF in pose 1.

In this study, we conducted molecular docking simulations to investigate the interaction between cisplatin and the MOF and observe their internal interactions, including the existing hydrogen bonds. For this purpose, we utilized Molegro Virtual Docker software. The structure of the MOF along with cisplatin was imported into the Molegro Virtual Docker software. After correcting and preparing the MOF structure prior to docking using the Preparation tab, the software was ready for docking execution. By accessing the Docking tab and Docking wizard, the Moledock se algorithm was selected along with the screening method, and the number of poses and runs was set to 5 and 10, respectively. In this simulation, 5 position calculations were considered. Upon completion of molecular docking, by visiting the poses tab, each of the 5 simulated poses could be observed. By examination of the energy of each pose, the best ones were selected. A more negative value indicates a better MOF-Cisplatin complex. According to the observed chart in Figure 2, the first pose with a MolDock score of −40.2498 was identified as the best pose. For further investigation of the docking output, MolegroViewer software was consulted. By accessing the Ligand Map tab and selecting Show interaction, the types of interactions could be observed for MOF-Cisplatin interactions. Upon examination of this pose, it is observed that cisplatin forms four hydrogen bonds with three MOF amino acids, Glu 139, Glu 60, and Glu 106, along with a steric interaction with Ser 55.

## 3 Results and Discussion

### 3.1 pH-Responsive Drug Release Simulations

Based on the molecular model structure proposed by Zhong et al. to investigate the release of aquatic cisplatin from the MOF, molecular dynamics simulations were performed at three different pHs (5.4, 6.4, 7.4).^45^ Several analyses were carried out to explore and elucidate the interactions between the drug and nanocarrier, as well as the drug release response to different pH conditions.^46^

#### 3.1.1 Total Energy Analysis

Total energy analyzes the amount of interaction of MOFs-Cis-Pt with the cancer cell membrane; a smaller amount of energy indicates a stronger interaction between the particles.^46^ Total energy arises from both electrostatic and van der Waals interactions between the two drug groups and the nanocarrier.^47^ Van der Waals interactions depend on structural stability as well as the size and mass of atoms and are governed by the Lennard-Jones equation.^48^ Electrostatic interactions, on the other hand, involve the calculation of charge differences between the nanocarrier and drug atoms, following Coulomb’s law.^49^ Increased van der Waals and electrostatic energies contribute to lower total energy, which in turn enhances drug and nanocarrier adsorption.^49^

**Figure 3:**
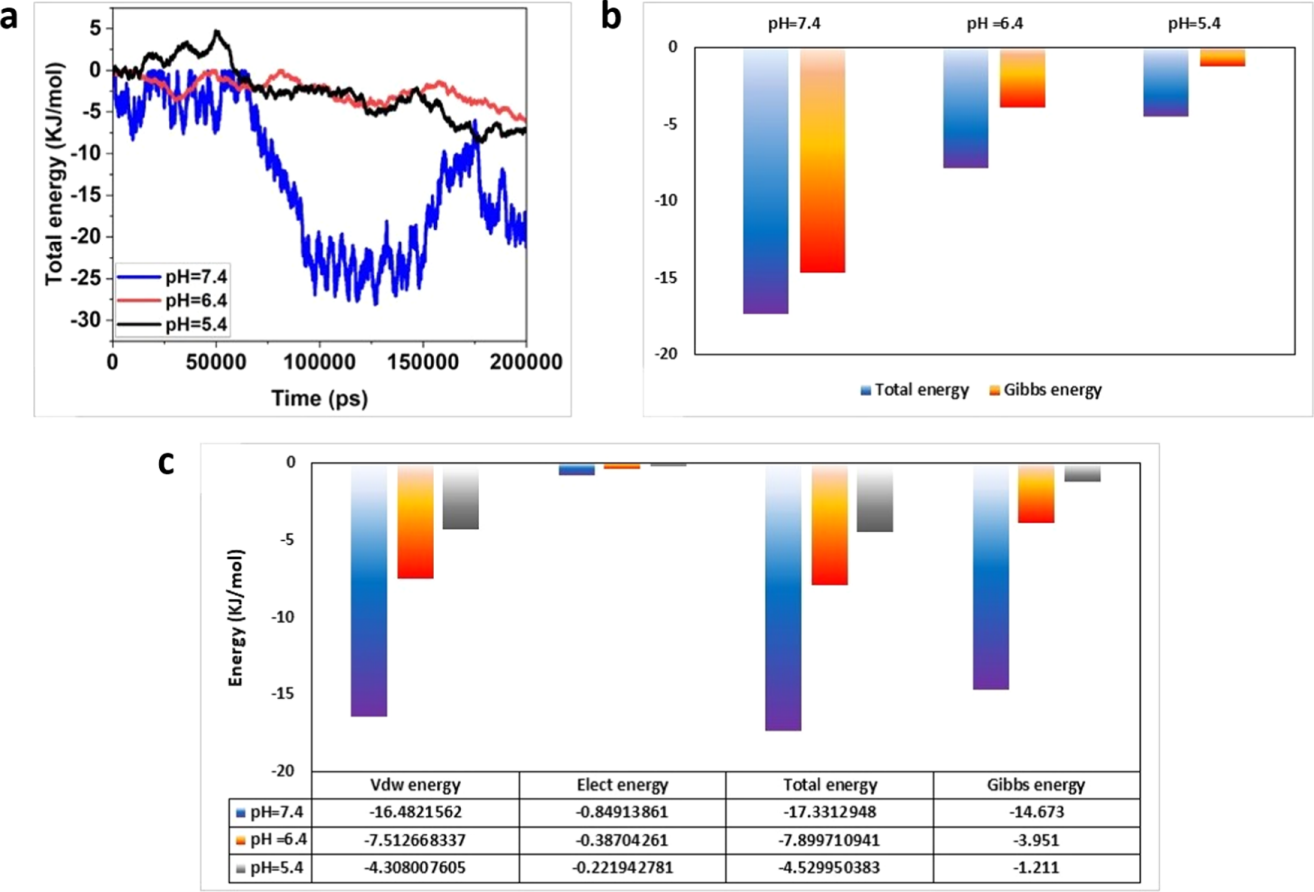
(a) Total energy across three pH levels. (b) Temporal plot of total energy at different pH levels, accompanied by charts illustrating the total energy and Gibbs energy at these levels. It is noteworthy that the Gibbs energy tends to become more negative at higher pH values. (c) Electrostatic and van der Waals energies relative to total and Gibbs energies at various pH levels.

The total energy between MOFs and Cis-Pt at pH 7.4, 6.4, and 5.4 was calculated in units of kJ/mol (Figure 3). The results indicate that the smallest total energy was observed for MOFs-Cis-Pt at pH 7.4, with a value of −17.1 kJ/mol. As the pH became more acidic, the total energy diagram showed a decrease in the adsorption between the drug and the nanocarrier. At pH 6.4, the total energy was −8.2 kJ/mol, and at pH 5.4, the total energy was −4.8 kJ/mol. These findings suggest that the system is more stable at neutral pH and that the adsorption of structures and charges between the drug and nanocarrier is more favorable compared to acidic conditions. The improved adsorption at neutral pH can be attributed to the presence of nonidentical charges on the drug and nanocarrier molecules. In acidic conditions, the system experiences repulsive electrostatic forces between similarly charged molecules, leading to an increase in total energy and a reduction in the intensity of adsorption between drug molecules and the nanocarrier. These results are supported by other dynamic simulations that highlight the vital role of vdW interactions in the stability of cisplatin loaded into carbon nanohorns and nanotubes.^50,51^

To achieve drug-nanocarrier conjugation, controlling the pH of the drug-nanocarrier system is crucial. According to the simulation and the graph in Figure 3, it is evident that higher pH values (7.4) result in more negative energy and thus greater stability compared to other pH levels. Additionally, it is noticeable that a more acidic pH (5.4) demonstrates the highest energy compared to others. This indicates repulsion between the drug carrier within the acidic pH range, corresponding to the time when the drug is being transferred to cancer cells, known as release. As the drug separates from the surface of the nanocarrier, it possesses the highest energy and consequently less stability.

Gibbs free energy was also calculated in absolute state; this energy is acquired from the umbrella sampling simulations.^44^ The smaller the Gibbs energy of the system, the more drugs and nanocarriers are attracted to each other.^52^ Consistent with the total energy results, the Gibbs free energy was found to be higher at neutral pH, indicating better stability and adsorption of drugs and nanocarriers. Additionally, a similar trend was observed for the increase in the Gibbs free energy as the system pH decreased. It is worth mentioning that the presented data were calculated as time averages. Li et al. observed a decrease in stability of the 2D metal–organic framework used as a drug delivery system (DDS) for cisplatin when the pH was lowered, particularly at pH 5.5 compared to pH 7.4.^53^

Additionally, the temporal plot of total energy is depicted, which illustrates the electrostatic and van der Waals energies plot relative to total and Gibbs energies (Figure 3).

#### 3.1.2 Radius of Gyration (*R*_g_) Analysis

**Figure 4:**
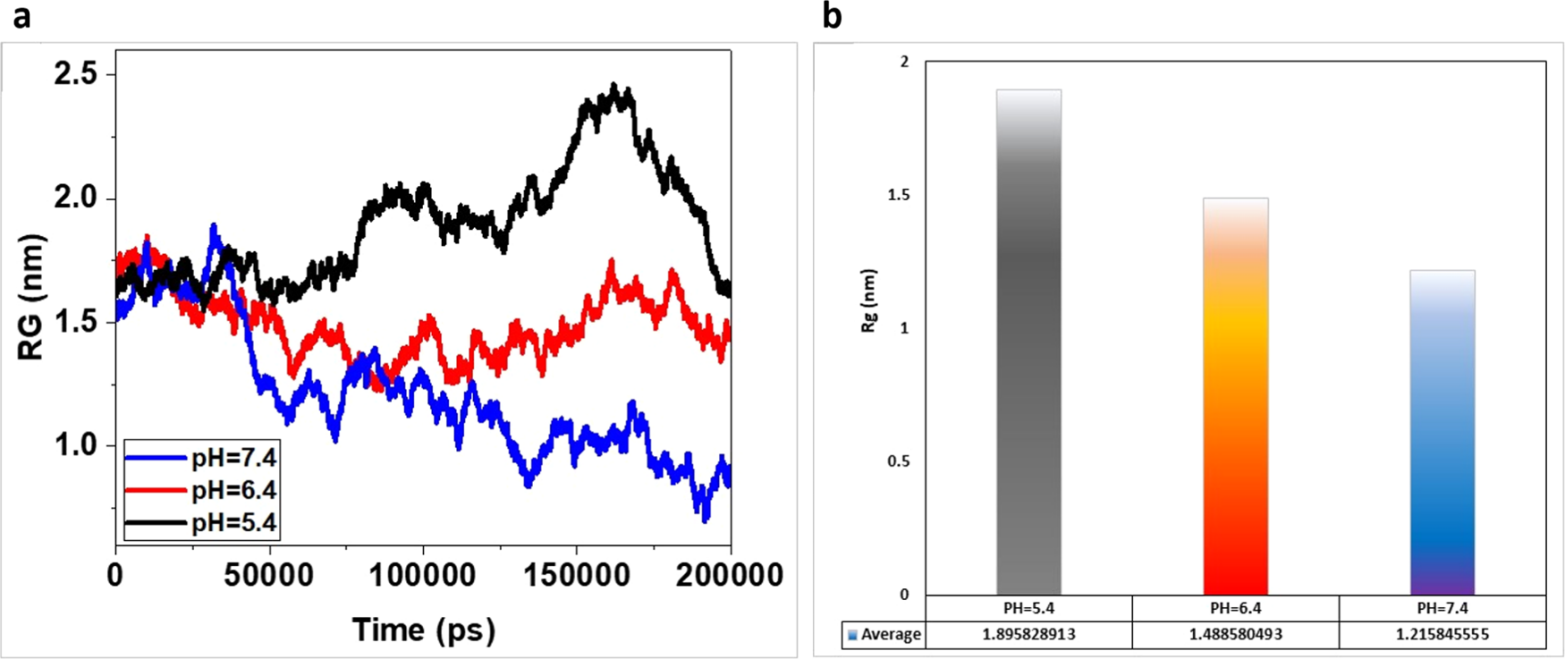
(a) Vertical axis represents the radius of gyration, while horizontal axis represents time in picoseconds. The plot displays the mean gyration radius at different pH levels. (b) Chart providing the average *R*_g_ at each pH and indicating that a smaller *R*_g_ is achieved at neutral pH.

*R*_g_ represents the root mean square of the nanoparticle’s distance from the center of mass, or center of gravity.^54,55^ It serves as a measure of the compactness and stability of structures in simulations. Thus, a smaller radius of the drug and nanocarriers’ spheres means better adsorption, stability, compactivity, and higher interactions between the nanocarrier and the drug.^54−56^Figure 4 illustrates the *R*_g_ diagram of groups including cisplatin and MOFs at pH 7.4, 6.4, and 5.4, considering *R*_g_ at different simulation times of the drug and nanocarriers as a hypothetical sphere with a variable radius.

The starting and ending points are critical in this analysis. In the graph, the starting point remains constant across different pH values, while the end points display varying ranges. At pH 7.4, the end point corresponds to a radius of gyration equal to 1.2 nm (Figure 4). A smaller *R*_g_ value at the end point signifies greater accumulation of drugs and drug molecules, indicating improved adsorption. This behavior was observed at pH 7.4. As the system progressed toward acidic pH levels, the end point of *R*_g_ increased and adsorption decreased.

#### 3.1.3 Radial Distribution Function (RDF) Analysis

**Figure 5:**
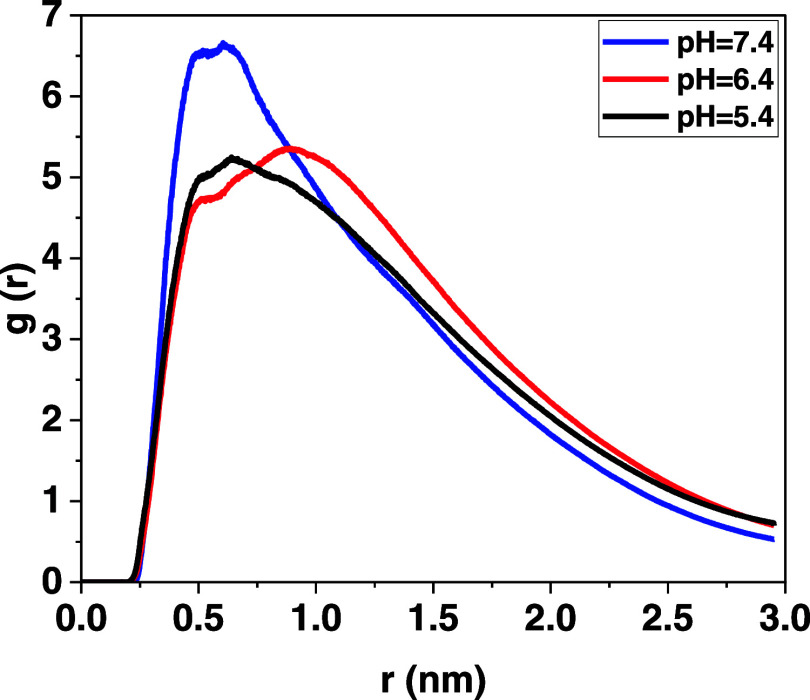
Diagram showing the radial distribution function. The highest value is related to molecular aggregation at pH 7.4.

In RDF analysis, we can observe the distribution of drug molecules (cisplatin) around MOFs and estimate the extent of their diffusion aggregation.^57,58^Figure 5 illustrates the RDF analysis graph for cisplatin and MOFs at different pH values.^59^ The RDF analysis provides insights into the adsorption criteria by evaluating the maximum value and visualizing the positions of the drug and nanocarrier within the simulation box.^60^ The vertical axis represents the maximum value in the graph.^57^ A higher maximum value in the graph indicates a stronger connection and closer proximity between the drug and nanocarrier molecules.^57^ The highest maximum value was observed at a neutral pH, which is 6.66. In comparison, both acidic systems have maximum values that are nearly the same. The maximum RDF value for pH 6.4 was 5.36, while for pH 5.4, it was 5.25. This suggests that less accumulation occurs at acidic pH levels and the optimal pH for adsorption, with a significant pH difference, is neutral. Another important parameter is the distance at which the highest RDF occurs. In the diagram, the maximum RDF was observed at 0.51 nm from the center of mass of the nanocarrier and drug groups for neutral pH. However, at acidic pHs (6.4 and 5.4), it occurred at distances of 0.52 and 0.56 nm, respectively. Therefore, at pH 7.4, a higher intensity of accumulation and distribution of nanocarrier and drug molecules was observed, indicating better adsorption and interaction between the drug and nanocarrier molecules.

In terms of distance, the graph shows its maximum value at 0.51 nm for pH 7.4, 0.52 nm for pH 5.4, and 0.56 nm for pH 6.4. The amplitude of the graph after the maximum point can indicate the presence of drug molecules at distances far from the nanocarrier. The larger the range of these graphs and the more flour they have, the more it shows the presence of drugs at distances greater than the 0.51, 0.52, and 0.56 nm points. The range of the graph is lower for neutral pH, and this shows that most of the drug molecules are around the nanocarrier and the presence of drugs is reduced at greater distances. Thus, this exhibits better adsorption under neutral pH conditions than adsorption in acidic conditions.

#### 3.1.4 Solvent Accessible Surface Area (SASA) Analysis

SASA reports the value of variation in the contact area and self-assembly around water and can be obtained by the following mathematical equation^44,47,54^
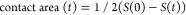


**Figure 6:**
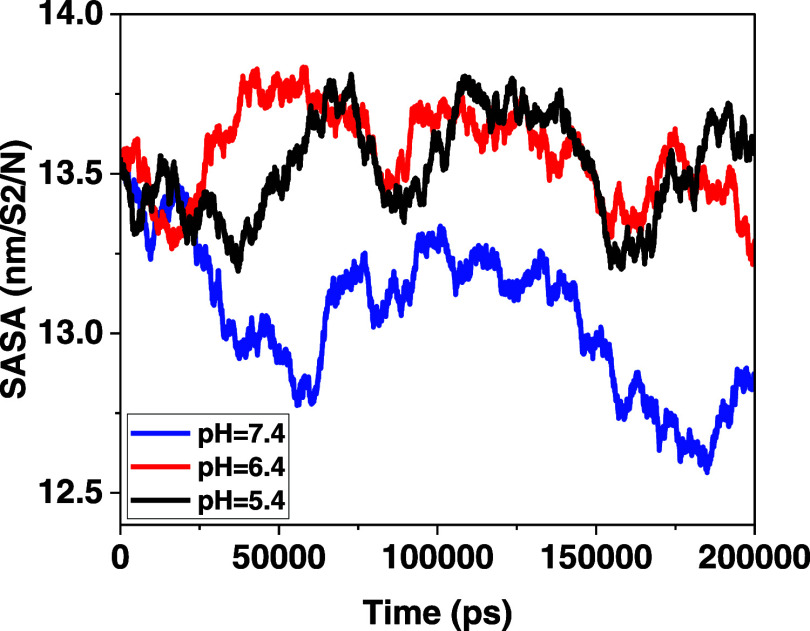
Vertical axis represents the area of the surface in cubic nanometers, and the horizontal axis represents the time in picoseconds.

Figure 6 presents a graph of the SASA analysis for cisplatin groups and MOFs at different pH levels. The reduced contact of the molecule’s surface with water indicates a stronger interaction and better adsorption between the drugs and nanocarriers. Specifically, at pH 7.4, the simulation results demonstrated a lower contact area of the molecular surface with water, particularly at the end points. This indicates a higher degree of adsorption. In contrast, acidic pHs exhibited a larger contact area with water, suggesting weaker adsorption.

Drug and nanocarrier distance increases at acidic pHs, indicating that drug release is a pH-responsive mechanism. Due to MOFs’ unique structure, they have proven to be useful for various applications, including ion transportation, water treatment, sensors in pharmacies, and energy storage.^61^ Therefore, many simulations and in vitro studies were conducted to observe MOF features such as delivering cisplatin to cancer cells to avoid resistance and side effect mechanisms.^34,61^ In 2014, He et al. reported that in an in vitro study, the codelivery of siRNA and cisplatin to ovarian cancer cells with nanoscale MOFs led to an increase in efficacy, as observed by DNA laddering, Annexin V staining, and viability assay.^34^ This study created the need to assess MOF’s properties as nanocarriers for cisplatin in cancer cell membranes. In this simulation, the total energy, which includes *R*_g_, was one of the geometric parameters available for analysis in the MD stage. Its larger number indicated a larger size of the drug delivery system; the average of *R*_g_ from larger to smaller was as follows: 5.4, 6.4, and 7.4. An important analysis in the study of the aggregation and density of nanoparticles and the drug delivery system is the study of RDF molecules in the simulation box. The higher the peak of this graph, the more concentrated and aggregated the accumulation of molecules. The maximization values in the RDF from largest to smallest based on the pH level were 7.4, 6.4, and 5.4. Therefore, the value of RDF at neutral pH had the highest accumulation, wheras this value was lower for acidic pHs, indicating better release and lesser accumulation. Another important factor in investigating the mechanism of system molecules and the box solvent interaction is the solvent accessible surface area analysis. This analysis is computed using the gmx sasa command. A lower SASA value indicates less system and solvent interference. Therefore, the molecules in the drug delivery system are more concentrated and integrated. Decreasing pH to acidic, its value increases. In short, the more acidic the environment is, the greater the amount of SASA, the greater the average radius of rotation, the higher the maximum amount of RDF, and the more positive the total energy.

These results indicate that as the end of the simulation time at acidic pHs approaches, the distance between the drug molecules and the nanocarrier increases, the drug release increases, and the level of molecular aggregation decreases. These cases are predictable due to the sensitivity of the drug release mechanism based on the functional groups present at the nanocarrier level and the positive charge of the drug. Gibbs free energy is a measure of the spontaneity of a reaction or phenomenon; therefore, the more negative this value is, the more spontaneous the reaction. And the more positive it is, the less inclined it is to react and interfere. This analysis was performed by using an umbrella sampling simulation. The value was the most negative for the neutral environment, which indicates the strong interaction between the drug and the nanocarrier, but with the increasing acidity of the simulation medium, its value also became more positive, indicating the tendency of molecules to separate and release the drug from the system.

#### 3.1.5 Root-Mean-Square Deviation (RMSD) Analysis

**Figure 7:**
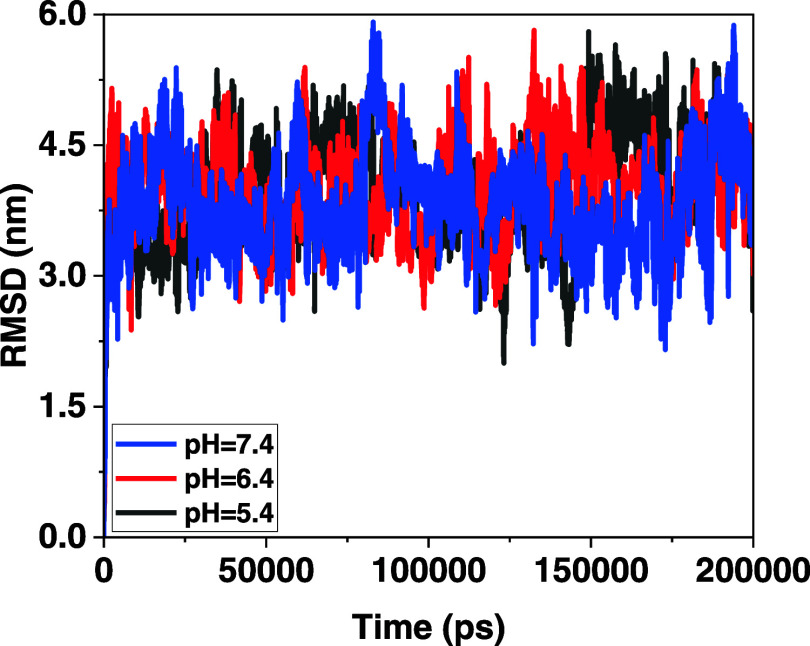
Root-mean-square deviation (RMSD) analysis plot for the MOF-cisplatin complex over time.

RMSD analysis assesses the deviation of fluctuations between two groups over a period. Certainly, the lower its value, the greater is the stability of the compound. In this simulation, the difference in fluctuations between the MOF and cisplatin is examined. Based on the RMSD plot presented in Figure 7, the MOF-cisplatin complex performed best at pH 7.4 and exhibited the highest stability. Indeed, the complex structure at pH 7.4 had the lowest RMSD compared to those of other pH values throughout the simulation. This indicates a greater stability during the simulation.

Moreover, RMSD fluctuations at pH 7.4 were also lower than other combinations and converged more rapidly toward a constant value compared to other combinations. This indicates that the complex structure at pH 7.4 quickly reaches a stable state. Subsequently, the complex structure at pH 6.4 demonstrates better stability and convergence compared to that of pH 5.4.

### 3.2 Interaction with Cancer Cell

**Figure 8:**
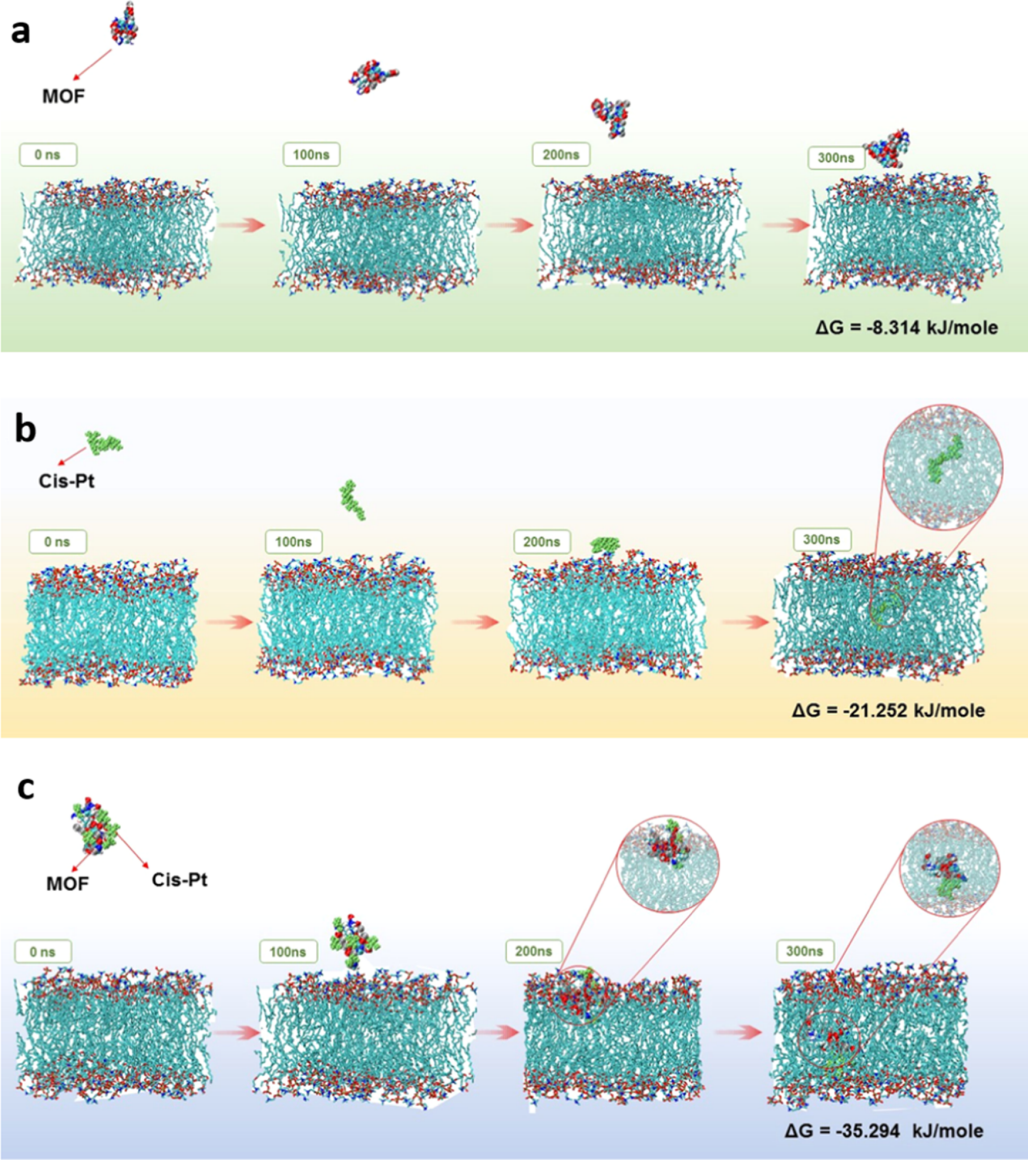
Molecular dynamics evaluation of the interaction between cancer cell membrane. (a) Metal–organic frameworks (MOFs), (b) Cisplatin (Cis-Pt), and (c) MOF-Cis-Pt. Gibbs Free energies. The best-penetrated nanoparticle is ordered as follows: (c, b, a).

Following the previous simulations, to investigate the toxicity of nanoparticles on the cancer cell membrane, we conducted an umbrella sampling simulation. The interaction of nanoparticles with cancerous membranes is a measure of the anticancer effect of these nanoparticles.^44^ Thereby, the Gibbs free energy is calculated in three modes: Cis-PT, MOFs-Cis-Pt, and MOFs. As indicated in Figure 8, numerical and schematic results obtained from molecular dynamics simulations provide insight into the study of the toxicity of these substances on cancerous masses. The results showed that Cis-Pt or MOF interactions with cancer cells alone were fewer spontaneous interactions. Gibbs free energy studies indicated that MOF-Cis-Pt interactions with the membrane were more spontaneous than others. Therefore, MOF-Cis-Pt has enough power to penetrate the surface of cancer cell membrane, while MOF is present only near the membrane and does not have enough power to penetrate the membrane.

## 4 Conclusions

In this study, molecular dynamics simulations were conducted to explore the role of MOFs in enhancing the delivery of cisplatin to cancer cells, providing fundamental insights into the synthesis and design of MOFs as drug delivery systems. During the molecular docking simulation to investigate the interactions between cisplatin and the MOF, three simulations were performed at different pH levels and in four stages, followed by umbrella sampling simulations. The analysis of total energy and Gibbs free energy demonstrated that neutral pH conditions promote higher adsorption of cisplatin, with the smallest total energy of −17.3 kJ/mol observed at pH 7.4 compared to −4.5 kJ/mol at pH 5.4. The radius of gyration (*R*_g_) of MOFs remained constant at the starting point across different pH levels, while at the end point, *R*_g_ decreased at pH 7.4 and increased at acidic pHs. This suggests that lower acidic pH levels facilitate increased accumulation of drug molecules, indicating higher adsorption levels. RDF analysis revealed significant findings regarding the value, distance, and contributions; pH 7.4 exhibited the highest value (6.66 nm), the shortest distance from the center of nanocarriers (0.51 nm), and the narrowest range of interaction between nanocarriers and drug molecules, indicating favorable interaction and adsorption. SASA analysis indicated that drug release is pH-dependent, with neutral pH demonstrating the least contact with water, leading to a reduced level of interference and improved integration between drug molecules and nanocarriers. RMSD analysis also indicated that the MOF-cisplatin complex structure exhibits a greater stability at pH 7.4. Based on the outputs and results of all analyses, especially the energy analysis, which serves as the fundamental basis for reporting the stability of structures, the simulated structure at pH 7.4 is selected as the most stable combination. Furthermore, the toxicity of the nanocarrier was evaluated by comparing Cis-Pt, MOF-Cis-Pt, and MOFs alone in simulations, revealing that MOF-Cis-Pt exhibited a higher potential for penetrating the cancerous cell membrane and displayed more spontaneous interactions.

## Abbreviations

DDS: drug delivery system
DNA: DNA
MOFs: metal–organic frameworks
RDF: radial distribution function
EM: energy minimization
NVT: canonical ensemble
NPT: isothermal–isobaric ensemble
MD: molecular dynamics
*R*_g_: radius of gyration
SASA: solvent accessible surface area

## References

[ref1] Tamaskovics B.; Haussmann J.; Karimi K.; Daum-Marzian M.; Gerber P. A.; Knapp F.; Scheckenbach K.; Bölke E.; Matuschek C.; Budach W. Recognizing cisplatin as a potential radiation recall trigger: case report and focused systematic review. Strahlenther. Onkol. 2023, 199, 611–620. 10.1007/s00066-023-02059-9. 36920507 PMC10281908

[ref2] Dasari S.; Tchounwou P. B. Cisplatin in cancer therapy: molecular mechanisms of action. Eur. J. Pharmacol. 2014, 740, 364–378. 10.1016/j.ejphar.2014.07.025. 25058905 PMC4146684

[ref3] Zoń A.; Bednarek I. Cisplatin in Ovarian Cancer Treatment-Known Limitations in Therapy Force New Solutions. Int. J. Mol. Sci. 2023, 24 (8), 7585 10.3390/ijms24087585. 37108749 PMC10146189

[ref4] Kartalou M.; Essigmann J. M. Recognition of cisplatin adducts by cellular proteins. Mutat. Res., Fundam. Mol. Mech. Mutagen. 2001, 478 (1–2), 1–21. 10.1016/S0027-5107(01)00142-7. 11406166

[ref5] Monneret C. Platinum anticancer drugs. From serendipity to rational design. Ann. Pharm. Fr. 2011, 69 (6), 286–295. 10.1016/j.pharma.2011.10.001. 22115131

[ref6] Wilmer A.; Gambihler S.; Delius M.; Brendel W. In vitro cytotoxic activity of lithotripter shock waves combined with adriamycin or with cisplatin on L1210 mouse leukemia cells. J. Cancer Res. Clin. Oncol. 1989, 115 (3), 229–234. 10.1007/BF00391694. 2753925 PMC12212472

[ref7] Aggarwal S. K.; Niroomand-Rad I. Effect of cisplatin on the plasma membrane phosphatase activities in ascites sarcoma-180 cells: a cytochemical study. J. Histochem. Cytochem. 1983, 31 (2), 307–317. 10.1177/31.2.6300219. 6300219

[ref8] Keller H. J.; Keppler B.; Schmähl D. Antitumor activity of cis-dihalogenobis(1-phenyl-1,3-butanedionato) titanium(IV) compounds against Walker 256 carcinosarcoma. A new class of antineoplastic agents. Arzneim.-Forsch. 1982, 32 (8), 806–807. 6890355

[ref9] Welsch C. W. Growth inhibition of rat mammary carcinoma induced by cis-platinum diamminodichloride-II. J. Natl. Cancer Inst. 1971, 47 (5), 1071–1078. 10.1093/jnci/47.5.1071. 5123334

[ref10] Kempf S. R.; Ivankovic S. Carcinogenic effect of cisplatin (cis-diammine-dichloroplatinum (II), CDDP) in BD IX rats. J. Cancer Res. Clin. Oncol. 1986, 111 (2), 133–136. 10.1007/BF00400751. 3084495 PMC12252877

[ref11] Beck D. J.; Brubaker R. R. Effect of cis-platinum(II)diamminodichloride on wild type and deoxyribonucleic acid repair deficient mutants of *Escherichia coli*. J. Bacteriol. 1973, 116 (3), 1247–1252. 10.1128/jb.116.3.1247-1252.1973. 4584807 PMC246480

[ref12] Fraval H. N.; Rawlings C. J.; Roberts J. J. Increased sensitivity of UV-repair-deficient human cells to DNA bound platinum products which unlike thymine dimers are not recognized by an endonuclease extracted from Micrococcus luteus. Mutat. Res., Fundam. Mol. Mech. Mutagen. 1978, 51 (1), 121–132. 10.1016/0027-5107(78)90014-3. 672924

[ref13] DeHaan R. D.; Yazlovitskaya E. M.; Persons D. L. Regulation of p53 target gene expression by cisplatin-induced extracellular signal-regulated kinase. Cancer Chemother. Pharmacol. 2001, 48 (5), 383–388. 10.1007/s002800100318. 11761456

[ref14] Jones E. V.; Dickman M. J.; Whitmarsh A. J. Regulation of p73-mediated apoptosis by c-Jun N-terminal kinase. Biochem. J. 2007, 405 (3), 617–623. 10.1042/BJ20061778. 17521288 PMC2267308

[ref15] Cuadrado A.; Lafarga V.; Cheung P. C.; Dolado I.; Llanos S.; Cohen P.; Nebreda A. R. A new p38 MAP kinase-regulated transcriptional coactivator that stimulates p53-dependent apoptosis. EMBO J. 2007, 26 (8), 2115–2126. 10.1038/sj.emboj.7601657. 17380123 PMC1852783

[ref16] Winograd-Katz S. E.; Levitzki A. Cisplatin induces PKB/Akt activation and p38(MAPK) phosphorylation of the EGF receptor. Oncogene 2006, 25 (56), 7381–7390. 10.1038/sj.onc.1209737. 16785992

[ref17] Duan X.; He C.; Kron S. J.; Lin W. Nanoparticle formulations of cisplatin for cancer therapy. WIREs Nanomed. Nanobiotechnol. 2016, 8 (5), 776–791. 10.1002/wnan.1390. PMC4975677 26848041

[ref18] Pourmadadi M.; Eshaghi M. M.; Rahmani E.; Ajalli N.; Bakhshi S.; Mirkhaef H.; Lasemi M. V.; Rahdar A.; Behzadmehr R.; Díez-Pascual A. M. Cisplatin-loaded nanoformulations for cancer therapy: A comprehensive review. J. Drug Delivery Sci. Technol. 2022, 77, 103928 10.1016/j.jddst.2022.103928.

[ref19] Jaiswal V. D.; Pangam D. S.; Dongre P. M. Biophysical study of cisplatin loaded albumin-gold nanoparticle and its interaction with glycans of gp60 receptor. Int. J. Biol. Macromol. 2023, 231, 123368 10.1016/j.ijbiomac.2023.123368. 36682660

[ref20] Ali R.; Aouida M.; Sulaiman A. A.; Madhusudan S.; Ramotar D. Can. Cisplatin Therapy Be Improved? Pathways That Can. Be Targeted. Int. J. Mol. Sci. 2022, 23 (13), 7241 10.3390/ijms23137241. 35806243 PMC9266583

[ref21] Zhu S.; Shanbhag V.; Wang Y.; Lee J.; Petris M. A Role for The ATP7A Copper Transporter in Tumorigenesis and Cisplatin Resistance. J. Cancer 2017, 8 (11), 1952–1958. 10.7150/jca.19029. 28819394 PMC5559955

[ref22] Li Z. H.; Zheng R.; Chen J. T.; Jia J.; Qiu M. The role of copper transporter ATP7A in platinum-resistance of esophageal squamous cell cancer (ESCC). J. Cancer 2016, 7 (14), 2085–2092. 10.7150/jca.16117. 27877224 PMC5118672

[ref23] Li Z. H.; Qiu M. Z.; Zeng Z. L.; Luo H. Y.; Wu W. J.; Wang F.; Wang Z. Q.; Zhang D. S.; Li Y. H.; Xu R. H. Copper-transporting P-type adenosine triphosphatase (ATP7A) is associated with platinum-resistance in non-small cell lung cancer (NSCLC). J. Transl. Med. 2012, 10, 21 10.1186/1479-5876-10-21. 22304828 PMC3296618

[ref24] Agnello L.; Tortorella S.; d’Argenio A.; Carbone C.; Camorani S.; Locatelli E.; Auletta L.; Sorrentino D.; Fedele M.; Zannetti A.; et al. Optimizing cisplatin delivery to triple-negative breast cancer through novel EGFR aptamer-conjugated polymeric nanovectors. J. Exp. Clin. Cancer Res. 2021, 40 (1), 239 10.1186/s13046-021-02039-w. 34294133 PMC8299618

[ref25] Han Y.; Wen P.; Li J.; Kataoka K. Targeted nanomedicine in cisplatin-based cancer therapeutics. J. Controlled Release 2022, 345, 709–720. 10.1016/j.jconrel.2022.03.049. 35367476

[ref26] Ashrafizadeh M.; Zarrabi A.; Bigham A.; Taheriazam A.; Saghari Y.; Mirzaei S.; Hashemi M.; Hushmandi K.; Karimi-Maleh H.; Zare E. N.; et al. Nano)platforms in breast cancer therapy: Drug/gene delivery, advanced nanocarriers and immunotherapy. Med. Res. Rev. 2023, 43, 2115–2176. 10.1002/med.21971. 37165896

[ref27] Jan N.; Madni A.; Khan S.; Shah H.; Akram F.; Khan A.; Ertas D.; Bostanudin M. F.; Contag C. H.; Ashammakhi N.; Ertas Y. N. Biomimetic cell membrane-coated poly(lactic-co-glycolic acid) nanoparticles for biomedical applications. Bioeng. Transl. Med. 2023, 8 (2), e10441 10.1002/btm2.10441. 36925703 PMC10013795

[ref28] Ertas Y. N.; Dorcheh K. A.; Akbari A.; Jabbari E. Nanoparticles for Targeted Drug Delivery to Cancer Stem Cells: A Review of Recent Advances. Nanomaterials 2021, 11 (7), 1755 10.3390/nano11071755. 34361141 PMC8308126

[ref29] Duverger E.; Balme S.; Bechelany M.; Miele P.; Picaud F. Natural payload delivery of the doxorubicin anticancer drug from boron nitride oxide nanosheets. Appl. Surf. Sci. 2019, 475, 666–675. 10.1016/j.apsusc.2018.12.273.

[ref30] Dehaghani M. Z.; Yousefi F.; Sajadi S. M.; Munir M. T.; Abida O.; Habibzadeh S.; Mashhadzadeh A. H.; Rabiee N.; Mostafavi E.; Saeb M. R. Theoretical Encapsulation of Fluorouracil (5-FU) Anti-Cancer Chemotherapy Drug into Carbon Nanotubes (CNT) and Boron Nitride Nanotubes (BNNT). Molecules 2021, 26 (16), 4920 10.3390/molecules26164920. 34443508 PMC8398462

[ref31] Katona G.; Sabir F.; Sipos B.; Naveed M.; Schelz Z.; Zupkó I.; Csóka I. Development of Lomustine and n-Propyl Gallate Co-Encapsulated Liposomes for Targeting Glioblastoma Multiforme via Intranasal Administration. Pharmaceutics 2022, 14 (3), 631 10.3390/pharmaceutics14030631. 35336006 PMC8950329

[ref32] Shen J. J.; Xue S. J.; Mei Z. H.; Li T. T.; Li H. F.; Zhuang X. F.; Pan L. M. Synthesis, characterization, and efficacy evaluation of a PH-responsive Fe-MOF@GO composite drug delivery system for the treating colorectal cancer. Heliyon 2024, 10 (6), e28066 10.1016/j.heliyon.2024.e28066. 38524612 PMC10957435

[ref33] Chen J.; Zhang Z.; Ma J.; Nezamzadeh-Ejhieh A.; Lu C.; Pan Y.; Liu J.; Bai Z. Current status and prospects of MOFs in controlled delivery of Pt anticancer drugs. Dalton Trans. 2023, 52 (19), 6226–6238. 10.1039/D3DT00413A. 37070759

[ref34] He C.; Lu K.; Liu D.; Lin W. Nanoscale metal-organic frameworks for the co-delivery of cisplatin and pooled siRNAs to enhance therapeutic efficacy in drug-resistant ovarian cancer cells. J. Am. Chem. Soc. 2014, 136 (14), 5181–5184. 10.1021/ja4098862. 24669930 PMC4210117

[ref35] Zhen J.; Ma F.; Yan J.; Lin R.; Yan M.; Wu Y. Synthesis of metal-organic framework hybrid nanocomposites based on MOFs@C3N4 with high selective separation ability for luteolin. Sep. Purif. Technol. 2024, 335, 126139 10.1016/j.seppur.2023.126139.

[ref36] Liu D.; Huxford R. C.; Lin W. Phosphorescent nanoscale coordination polymers as contrast agents for optical imaging. Angew. Chem., Int. Ed. 2011, 50 (16), 3696–3700. 10.1002/anie.201008277. PMC3432504 21416573

[ref37] Rieter W. J.; Taylor K. M.; An H.; Lin W.; Lin W. Nanoscale metal-organic frameworks as potential multimodal contrast enhancing agents. J. Am. Chem. Soc. 2006, 128 (28), 9024–9025. 10.1021/ja0627444. 16834362 PMC2556368

[ref38] Qi X.; Shen N.; Al Othman A.; Mezentsev A.; Permyakova A.; Yu Z.; Lepoitevin M.; Serre C.; Durymanov M. Metal-Organic Framework-Based Nanomedicines for the Treatment of Intracellular Bacterial Infections. Pharmaceutics 2023, 15 (5), 1521 10.3390/pharmaceutics15051521. 37242762 PMC10220673

[ref39] Masoudifar R.; Pouyanfar N.; Liu D.; Ahmadi M.; Landi B.; Akbari M.; Moayeri-Jolandan S.; Ghorbani-Bidkorpeh F.; Asadian E.; Shahbazi M.-A. Surface engineered metal-organic frameworks as active targeting nanomedicines for mono- and multi-therapy. Appl. Mater. Today 2022, 29, 101646 10.1016/j.apmt.2022.101646.

[ref40] Shen X.; Pan Y.; Sun Z.; Liu D.; Xu H.; Yu Q.; Trivedi M.; Kumar A.; Chen J.; Liu J. Design of Metal-Organic Frameworks for pH-Responsive Drug Delivery Application. Mini Rev. Med. Chem. 2019, 19 (20), 1644–1665. 10.2174/1389557519666190722164247. 31880236

[ref41] Guillen S. G.; Parres-Gold J.; Ruiz A.; Lucsik E.; Dao B.; Hang T. K. L.; Chang M.; Garcia A. O.; Wang Y.; Tian F. pH-Responsive Metal–Organic Framework Thin Film for Drug Delivery. Langmuir 2022, 38 (51), 16014–16023. 10.1021/acs.langmuir.2c02497. 36516863 PMC9798862

[ref42] Saeb M. R.; Rabiee N.; Mozafari M.; Verpoort F.; Voskressensky L. G.; Luque R. Metal-Organic Frameworks (MOFs) for Cancer Therapy. Materials 2021, 14 (23), 7277 10.3390/ma14237277. 34885431 PMC8658485

[ref43] Karplus M.; McCammon J. A. Molecular dynamics simulations of biomolecules. Nat. Struct. Biol. 2002, 9 (9), 646–652. 10.1038/nsb0902-646. 12198485

[ref44] Maleki R.; Khedri M.; Malekahmadi D.; Mohaghegh S.; Jahromi A. M.; Shahbazi M.-A. Simultaneous doxorubicin encapsulation and in-situ microfluidic micellization of bio-targeted polymeric nanohybrids using dichalcogenide monolayers: A molecular in-silico study. Mater. Today Commun. 2021, 26, 101948 10.1016/j.mtcomm.2020.101948.

[ref45] Zhong R.-Q.; Zou R.-Q.; Xu Q. Microporous metal-organic framework zinc (II) imidazole-4, 5-dicarboxylate: Four-fold helical structure and strong fluorescent emission. Microporous Mesoporous Mater. 2007, 102 (1–3), 122–127. 10.1016/j.micromeso.2006.12.041.

[ref46] Zeng S.; Quan X.; Zhu H.; Sun D.; Miao Z.; Zhang L.; Zhou J. Computer Simulations on a pH-Responsive Anticancer Drug Delivery System Using Zwitterion-Grafted Polyamidoamine Dendrimer Unimolecular Micelles. Langmuir 2021, 37 (3), 1225–1234. 10.1021/acs.langmuir.0c03217. 33417464

[ref47] Khedri M.; Rezvantalab S.; Maleki R.; Rezaei N. Effect of ligand conjugation site on the micellization of Bio-Targeted PLGA-Based nanohybrids: A computational biology approach. J. Biomol. Struct. Dyn. 2022, 40 (10), 4409–4418. 10.1080/07391102.2020.1857840. 33336619

[ref48] Semironi D. T.; Azimian A. R. Molecular dynamics simulation of liquid–vapor phase equilibrium by using the modified Lennard-Jones potential function. Heat Mass Transfer 2010, 46 (3), 287–294. 10.1007/s00231-009-0566-x.

[ref49] Sohrabi S.; Khedri M.; Maleki R.; Moraveji M. K. Molecular engineering of the last-generation CNTs in smart cancer therapy by grafting PEG–PLGA–riboflavin. RSC Adv. 2020, 10 (67), 40637–40648. 10.1039/D0RA07500K 10.1039/D0RA07500K. 35519185 PMC9057702

[ref50] Khatti Z.; Hashemianzadeh S. M.; Shafiei S. A. A Molecular Study on Drug Delivery System Based on Carbon Nanotube Compared to Silicon Carbide Nanotube for Encapsulation of Platinum-Based Anticancer Drug. Adv. Pharm. Bull. 2018, 8 (1), 163–167. 10.15171/apb.2018.020. 29670852 PMC5896391

[ref51] Almeida E. R.; De Souza L. A.; De Almeida W. B.; Dos Santos H. F. Chemically Modified Carbon Nanohorns as Nanovectors of the Cisplatin Drug: A Molecular Dynamics Study. J. Chem. Inf. Model. 2020, 60 (2), 500–512. 10.1021/acs.jcim.9b00775. 31738559

[ref52] Maleki R.; Khoshoei A.; Ghasemy E.; Rashidi A. Molecular insight into the smart functionalized TMC-Fullerene nanocarrier in the pH-responsive adsorption and release of anti-cancer drugs. J. Mol. Graphics Modell. 2020, 100, 107660 10.1016/j.jmgm.2020.107660. 32659627

[ref53] Li Y.; Gao Z.; Chen F.; You C.; Wu H.; Sun K.; An P.; Cheng K.; Sun C.; Zhu X.; Sun B. Decoration of Cisplatin on 2D Metal–Organic Frameworks for Enhanced Anticancer Effects through Highly Increased Reactive Oxygen Species Generation. ACS Appl. Mater. Interfaces 2018, 10 (37), 30930–30935. 10.1021/acsami.8b12800. 30183247

[ref54] Rezvantalab S.; Moraveji M. K.; Khedri M.; Maleki R. An insight into the role of riboflavin ligand in the self-assembly of poly(lactic-co-glycolic acid)-based nanoparticles – a molecular simulation and experimental approach. Soft Matter 2020, 16 (22), 5250–5260. 10.1039/D0SM00203H. 32458880

[ref55] Alimohammadi E.; Nikzad A.; Khedri M.; Rezaian M.; Jahromi A. M.; Rezaei N.; Maleki R. Potential treatment of Parkinson’s disease using new-generation carbon nanotubes: a biomolecular in silico study. Nanomedicine 2021, 16 (3), 189–204. 10.2217/nnm-2020-0372. 33502255

[ref56] Alimohammadi E.; Maleki R.; Akbarialiabad H.; Dahri M. Novel pH-responsive nanohybrid for simultaneous delivery of doxorubicin and paclitaxel: an in-silico insight. BMC Chem. 2021, 15 (1), 11 10.1186/s13065-021-00735-4. 33573669 PMC7879683

[ref57] Maleki R.; Afrouzi H. H.; Hosseini M.; Toghraie D.; Rostami S. Molecular dynamics simulation of Doxorubicin loading with N-isopropyl acrylamide carbon nanotube in a drug delivery system. Comput. Methods Programs Biomed. 2020, 184, 105303 10.1016/j.cmpb.2019.105303. 31901633

[ref58] Yang W.; Xia X.; Liu X.; Zhang S. Interlayer structure and dynamic properties of CTMAB-montmorillonite: experiment and molecular dynamics. RSC Adv. 2023, 13 (19), 13324–13336. 10.1039/D3RA01834B. 37143701 PMC10152231

[ref59] Xu H.; Li Z.; Zhang Z.; Liu S.; Shen S.; Guo Y. High-Accuracy Neural Network Interatomic Potential for Silicon Nitride. Nanomaterials 2023, 13 (8), 1352 10.3390/nano13081352. 37110937 PMC10145480

[ref60] Abbaspour M.; Fotourechi F.; Akbarzadeh H.; Salemi S. Investigation of small inhibitor effects on methane hydrate formation in a carbon nanotube using molecular dynamics simulation. RSC Adv. 2023, 13 (10), 6800–6807. 10.1039/D2RA06518E. 36865572 PMC9971844

[ref61] Mashhadzadeh A. H.; Taghizadeh A.; Taghizadeh M.; Munir M. T.; Habibzadeh S.; Salmankhani A.; Stadler F. J.; Saeb M. R. Metal–Organic Framework (MOF) through the Lens of Molecular Dynamics Simulation: Current Status and Future Perspective. J. Compos. Sci. 2020, 4 (2), 75 10.3390/jcs4020075.

